# Feasibility and Safety of a Single‐Session of Transcutaneous Cervical Magnetic Stimulation, taVNS, and iTBS on Heart Rate Variability, Safety, and Pain Modulation

**DOI:** 10.1111/ejn.70370

**Published:** 2025-12-27

**Authors:** Tiago da Silva Lopes, Larissa Conceição Dias Lopes, Thaiany Pedrozo Campos Antunes, Isabela Rocha Fernandes, Márcia Midori Morimoto, Gustavo Pinheiro, Erick Dario León Bueno de Camargo, Bruna Ferreira Nonato, Maria Sophia Cantisani, Karina Rabello Casali, Pedro Montoya, Yossi Zana, Abrahão Fontes Baptista

**Affiliations:** ^1^ Center for Mathematics, Computing and Cognition Federal University of ABC São Paulo Brazil; ^2^ NAPEN Network (Nucleus of Assistance, Research, and Teaching in Neuromodulation) São Paulo São Paulo Brazil; ^3^ Municipal University of São Caetano do Sul (USCS) São Caetano do Sul São Paulo Brazil; ^4^ Center of Engineering, Modeling and Applied Social Sciences Federal University of ABC Santo André Brazil; ^5^ Institute of Science and Technology, Federal University of São Paulo, São José dos Campos, Brazil São Paulo Brazil; ^6^ Research Institute of Health Sciences (IUNICS) and Balearic Islands Health Research Institute (IdISBa) University of the Balearic Islands (UIB) Palma Spain; ^7^ School of Physiotherapy Federal University of Rio de Janeiro Rio de Janeiro Brazil

**Keywords:** autonomic nervous system, descending pain control, neuromodulation, neuroscience

## Abstract

The autonomic nervous system (ANS) plays a crucial role in maintaining homeostasis, and its dysfunction is linked to numerous clinical conditions, including chronic pain. Neuromodulatory interventions such as transcutaneous auricular vagus nerve stimulation (taVNS), transcutaneous cervical magnetic stimulation (tCMS), and intermittent theta burst stimulation (iTBS) have been investigated for their potential to modulate autonomic responses and pain perception. However, the efficacy and safety of these techniques remain unclear. This study aimed to evaluate the feasibility and safety of a single session of neuromodulatory stimulation in modulating autonomic function and pain processing in healthy individuals. A double‐blind, randomized, crossover clinical trial was conducted with 22 healthy participants, each undergoing four intervention sessions (taVNS, tCMS, iTBS, and Sham‐taVNS) in randomized order, with a washout period of at least 36 h between sessions. Heart rate variability (HRV) and conditioned pain modulation (CPM) were assessed pre‐ and post‐intervention using a Polar H10 cardiac sensor and a digital pressure algometer. Adverse effects were recorded immediately after each session. No statistically significant differences were observed in HRV or CPM outcomes across active stimulation conditions when compared to Sham. Among the techniques evaluated, tCMS presented the most favorable safety profile, with fewer reported adverse effects relative to iTBS and taVNS. The absence of significant modulation effects suggests that a single session may be insufficient to induce detectable changes in autonomic or pain processing. However, the tolerability and safety of tCMS indicate its potential for future research involving repeated sessions and clinical populations.

## Graphical abstract

tCMS showed the most favorable safety profile, with fewer adverse effects than taVNS and iTBS. No significant differences were observed in heart rate variability or pain modulation between the stimulation groups and the Sham stimulation group.

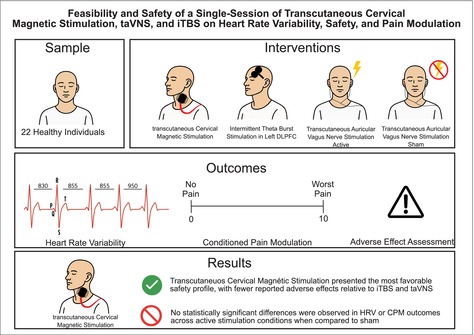

**Abbreviations**
ANS: autonomic nervous system
BMI: body mass index
CPM: conditioned pain modulation
DLPFC: dorsolateral prefrontal cortex
GEEs: generalized estimating equations
HFs: high frequencies
HRV: heart rate variability
IPAQ‐SF: International Physical Activity Questionnaire short‐form
iTBS: intermittent theta burst stimulation
LFs: low frequencies
pNN50: percentage of successive R–R intervals that differ by more than 50 ms
RMT: resting motor threshold
RMSSDs: root mean square of successive differences
rTMS: repetitive transcranial magnetic stimulation
SDNN: standard deviation of all R–R intervals
taVNS: transcutaneous auricular vagus nerve stimulation
tCMS: transcutaneous cervical magnetic stimulation
VLFs: very low frequencies

## 1 Introduction

The autonomic nervous system (ANS) plays a central role in maintaining bodily homeostasis by regulating vital functions such as heart rate, blood pressure, gastrointestinal motility, and temperature regulation (Gibbons 2019). In addition to these physiological functions, the ANS plays a key role in clinical conditions such as chronic pain, cognitive disorders, and motor deficits (Arslan and Ünal Çevik 2022; Blase et al. 2021; Sharabi et al. 2021). Given its multifaceted role, the ANS is a promising target for therapeutic interventions, especially in conditions characterized by imbalances between sympathetic and parasympathetic activity. Chronic pain, for example, is often associated with reduced parasympathetic activity and decreased heart rate variability (HRV) (Tracy et al. 2016), suggesting diminished vagal tone and impaired autonomic regulation related to pain modulation (Van Den Houte et al. 2018).

The modulation of autonomic function by neuromodulatory techniques, such as transcutaneous vagus nerve stimulation (taVNS) at the auricular branch of the vagus nerve, and repetitive transcranial magnetic stimulation (rTMS) on the left dorsolateral prefrontal cortex (DLPFC) has been described in healthy and sick individuals (Huang et al. 2022; Iseger et al. 2020; Jensen et al. 2022; Kang et al. 2024; Pinto et al. 2023; Sagui et al. 2024; Schmaußer et al. 2024; Zou et al. 2024). taVNS involves the non‐invasive electrical stimulation of the auricular branch of the vagus nerve through electrodes placed on the ear, activating afferent vagal fibers and influencing central autonomic networks (Clancy et al. 2014; Machetanz et al. 2021). rTMS, in contrast, uses rapidly changing magnetic fields applied over cortical targets to induce electric currents that modulate neuronal excitability (Huang et al. 2022; Iseger et al. 2020; Jensen et al. 2022; Kang et al. 2024; Pinto et al. 2023; Sagui et al. 2024; Schmaußer et al. 2024; Zou et al. 2024). A patterned form of rTMS, known as intermittent theta burst stimulation (iTBS), delivers bursts of three pulses at 50 Hz repeated at 5 Hz, mimicking natural theta rhythms and producing excitatory effects on cortical circuits (Blumberger et al. 2018; Chen et al. 2020; M. Zhang, Li, et al. 2024).

In healthy individuals, data show that taVNS modulates HRV in both the time and frequency domains, increasing HF power, RMSSD, and pNN50, decreasing the LF/HF ratio, and thus reflecting enhanced parasympathetic activity (Clancy et al. 2014; Machetanz et al. 2021). Similarly, this pattern was also observed in individuals with irritable bowel syndrome who underwent repeated taVNS sessions, and this change was accompanied by a notable reduction in their pain intensity (Liu et al. 2024). In turn, a recent systematic review showed that excitatory rTMS, such as iTBS, on the DLPFC was also able to modulate parasympathetic activity, increasing RMSSD and HF and reducing the LF/HF ratio, which reinforces its potential in autonomic regulation (H. Lee, Lee, et al. 2023).

Beyond its auricular portion, the vagus nerve can also be stimulated through its cervical branch via electrical or magnetic methods, although its effects on the ANS remain uncertain. Transcutaneous cervical magnetic stimulation (tCMS) applies magnetic pulses over the cervical region to influence peripheral and central autonomic pathways through stimulation of cervical neural structures, including branches of the vagus nerve (H. Zhang, Zhao, et al. 2024). In this context, tCMS has been employed in clinical and experimental settings due to the presence of key cervical nervous structures, such as the phrenic nerve, stellate ganglion, carotid baroreceptor, and the cervical branch of the vagus nerve (Adler et al. 2011; Boyle et al. 2022; Gmitrov 2022; Markman et al. 2022; Similowski et al. 1989). The cervical branch of the vagus nerve is a frequent target for modulating ANS activity; however, a recent clinical study in patients with traumatic brain injury found no significant changes in heart rate or blood pressure following tCMS, despite improvements in visuospatial, executive, and memory‐related tasks (H. Zhang, Zhao, et al. 2024). In contrast, another study using transcutaneous cervical vagus nerve electrical stimulation reported enhanced auditory and visual performance in individuals with lower sensory abilities, as well as an increase in HRV (Jigo et al. 2024). These divergent findings suggest that the mechanisms of action on the ANS may differ, despite both methods having anatomical plausibility for autonomic function modulation.

The safety of autonomic stimulation is another important issue to consider. The presence of adverse events is crucial to understand and evaluate, especially when a new apparatus is used, such as tCMS. In well‐established neuromodulatory techniques, such as iTBS and taVNS, the safety of stimulation has been documented (Daoud et al. 2024; Kim et al. 2022; Lench et al. 2023; Shen and Fang 2024). For example, taVNS studies reported mild and transient adverse effects, such as ear pain, headache, and tingling, with no significant increase in severe adverse events compared to controls (Kim et al. 2022; Lench et al. 2023). Similarly, iTBS in DLPFC has been applied in various clinical conditions, including stroke (Daoud et al. 2024) and psychiatric disorders (Li et al. 2024; Shen and Fang 2024; M. Zhang, Li, et al. 2024), and was considered safe with mild adverse effects reported, such as sneezing, headache, facial twitching, and facial/eye pain symptoms, which ceased a few times after the stimulation stopped. In contrast, tCMS has been less studied, with emerging evidence suggesting minimal side effects, such as transient neck stiffness, as observed in a recent feasibility study (H. Zhang, Zhao, et al. 2024). However, concerns remain regarding the co‐stimulation of non‐vagal fibers and the potential for cardiac impacts, particularly given the proximity of other neural and vascular structures in the cervical region (Chaudhary et al. 2020; M. Lee, Gan, et al. 2023).

The primary outcome to assess the effectiveness of tCMS was the HRV, quantified through both time (normal‐to‐normal R–R intervals—SDNN—and the root mean square of successive differences [RMSSDs]) and frequency (the spectral power of low and high‐frequency components) domains. Based on prior evidence that noninvasive neuromodulation can enhance parasympathetic tone (Clancy et al. 2014; Machetanz et al. 2021; H. Lee, Lee, et al. 2023), we hypothesized that a single session of tCMS would increase SDNN, RMSSD, and HF power, decrease the LF/HF ratio, and thereby reflect a shift toward parasympathetic dominance, as it occurs with the application of the other techniques. Thus, this study first aimed to evaluate the effectiveness and safety of a single session of tCMS delivered via a novel device designed to modulate the cervical branch of the vagus nerve. Second, this study aimed to compare tCMS effects with previously known techniques to modulate ANS activity, such as taVNS and iTBS, and their impact on opioid endogenous modulation in healthy individuals.

## 2 Method

### 2.1 Study Design and Participants

This double‐blind randomized crossover clinical trial (NCT06689345) was conducted in the Applied Neuromechanics Laboratory at the Federal University of ABC, São Bernardo do Campo, Brazil. Inclusion criteria were pain‐free adults of both sexes, between 18 and 64 years old, and healthy. The exclusion criteria were cardiovascular disease, musculoskeletal disease, or other pain syndrome diagnosis.

### 2.2 Sample Size

The sample size of 16 individuals was calculated using G*Power 3.1.9.7 software, considering an effect size of 0.4 for autonomic stimulation by neuromodulatory techniques over HRV. The alpha level was set at 5%, the study power at 80%, and four baseline data acquisition blocks were obtained before each stimulation session, followed by one post‐stimulation measurement. There was a correlation among measures of 0.5 and a non‐sphericity correction of 1. We increased the sample size to 22 individuals to prevent problems with data loss.

### 2.3 Randomization, Blinding, and Intervention

There were four types of intervention: (a) tCMS; (b) iTBS; (c) taVNS; and (d) Sham‐taVNS. All individuals received the four interventions in a balanced order with a washout period of at least 36 h. Allocation to a specific treatment order was random. One staff member was designated to generate an allocation table using the www.randomizer.org website and store it in sequentially numbered opaque envelopes.

**FIGURE 1:**
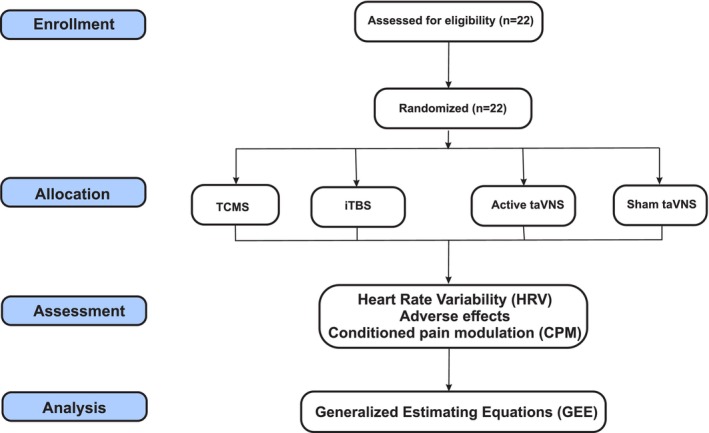
Flowchart of the study based on CONSORT criteria.

During the interventions, all individuals were comfortably seated in a chair with their backs to the equipment. They did not have access to any information related to the stimulation configuration. The tCMS was delivered on the cervical branch of the afferent vagus nerve with a cervical (local) motor threshold intensity. The coil (Cool Twin B‐46, Magventure, Denmark) was positioned 2 cm below the angle of the jaw along the anterior border of the right sternocleidomastoid muscle. The parameters used were 25 Hz, 1 s on, and 1 s off; 1550 pulses per session with a total duration of 2 min. The iTBS was delivered at the left dorsolateral prefrontal cortex with an intensity of 120% of the resting motor threshold (RMT). The parameters were the triplet 50 Hz bursts, an interpulse interval of 20 ms, repeated at 5 Hz; 2 s on and 8 s off; 600 pulses per session with a total duration of 3 min and 8 s (M. Zhang, Li, et al. 2024). The left dorsolateral prefrontal cortex was estimated using the beamF3 method (Mir‐Moghtadaei et al. 2015). All the magnetic stimulations were delivered using a magnetic stimulator (Magventure R30 with Mag Option, Denmark). The taVNS was delivered using a biphasic electrical stimulator (EL‐608, NKL, Brazil), with electrodes allocated at the bilateral concha cymba, the sensory threshold amplitude, with 25 Hz, 30 s on, 30 s off, and a total duration of 30 min per session (Thompson et al. 2021). Finally, the taVNS‐Sham was mounted similarly to the active taVNS group but only lasted 20 s. Both individuals and staff evaluators were blind to the allocation group, and all individuals were instructed not to discuss their interventions with other participants or staff members (Figure 1).

### 2.4 Measurements

Initially, sociodemographic data on occupation status, age, and sex were collected. Next, a portable stadiometer (Balmak, Brazil) was used to measure standing height, and weight was determined using a portable weighing scale (Wiso, USA). The body mass index (BMI) was calculated for all individuals $\mathrm{BMI}=\text{weight}\;\left(\mathrm{kg}\right)/\text{height}\left(\mathrm{cm}\right)$. The physical activity level was measured using the International Physical Activity Questionnaire short‐form (IPAQ‐SF), a standardized tool for measuring physical activity levels in adults, covering activities performed over the last 7 days. The IPAQ‐SF assesses the duration and frequency of walking, and moderate and vigorous activities, estimating total physical activity (Matsudo et al. 2001). These sociodemographic and lifestyle variables were collected to provide a comprehensive characterization of the sample and to control for potential confounding factors known to influence ANS activity and HRV. The individuals were then asked to lie down and relax for 5 min to stabilize their heart rate and blood pressure. The room temperature was maintained at 23°C, and the brightness was reduced. Throughout the relaxation period, individuals were instructed to keep their eyes open. Blood pressure was measured using a digital monitor (G‐Tech, Brazil). Breathing and heart rate were measured simultaneously with individuals lying down with their eyes closed. The breathing was monitored using a respiration monitor (Respiration Belt RM‐204, IWorks, USA) positioned approximately 2 cm below the xiphoid process, and the individuals were instructed to breathe normally throughout the measurements. Heart rate was measured for 6 min using a Polar H10 cardiac sensor (Polar Electro, Finland) positioned on the xiphoid process (Schaffarczyk et al. 2022).

### 2.5 HRV Measures

HRV was estimated from the R–R intervals and processed using the Kubios software (Tarvainen et al. 2014). The Kubios software includes an automated artifact correction algorithm that identifies and corrects ectopic beats and other abnormal R‐R intervals. In addition, all recordings were manually inspected by an expert to verify the accuracy of the automated correction and to ensure that any remaining artifacts were appropriately handled. In the time domain, the standard deviation of all R–R intervals (SDNN) was calculated to reflect overall HRV (“Heart Rate Variability. Standards of Measurement, Physiological Interpretation, and Clinical Use. Task Force of the European Society of Cardiology and the North American Society of Pacing and Electrophysiology,” 1996), and the RMSSDs were calculated to reflect vagal tone (Trimmel et al. 2015). In the frequency domain, the stationarity of the signal was evaluated by an expert and confirmed using the following equation: $\left[\mathrm{VLF}\;\text{power}/\left(\mathrm{LF}\;\text{power}+\mathrm{HF}\;\text{power}\right)\right]\times 100$ where the VLF power should be less than 80% of the sum of LF and HF powers. The total power HRV, which reflects the total power of HF, LF, and VLF waves, was calculated. In addition, the mean spectral power of low frequencies (LFs) and high frequencies (HFs), in the range of 0.04–0.15 and 0.15–0.40 Hz, respectively, was also calculated. These frequency bands reflect the influence of both sympathetic and parasympathetic branches, and vagal tone, respectively (“Heart Rate Variability. Standards of Measurement, Physiological Interpretation, and Clinical Use. Task Force of the European Society of Cardiology and the North American Society of Pacing and Electrophysiology,” 1996). Additionally, the autonomic response of normalized LF and HF components to neuromodulatory interventions was assessed by calculating the ratio of the normalized power spectrum after the intervention to that at baseline.

### 2.6 Conditioned Pain Modulation (CPM)

The CPM was assessed in three steps: (a) The mechanical pain threshold was measured using a pressure algometer (SPtech, Medeor MedTech, Brazil), positioned on the right forearm approximately 7.5 cm from the distal wrist crease. Gradual pressure was applied at about 1 kg/min perpendicularly to the forearm until the individual reported the sensation as painful. This procedure was called the test stimulus. (b) An ischemic pressure was applied to the left arm using a manual sphygmomanometer (Welch Allyn, USA). The bottom edge of the sphygmomanometer was positioned approximately 3 cm above the cubital fossa, and pressure was gradually increased to about 270 mmHg. This pressure was maintained until the individual reported a moderate pain level of 4/11 on the visual analog scale. This procedure was called the conditioned test stimulus. (c) The test stimulus was measured again during and 5 min after the conditioned test stimulus (Kennedy et al. 2016). The CPM was calculated for all individuals as $\left[\left(\mathrm{fTS}-\mathrm{sTS}\right)/\mathrm{fTS}\right]\times 100$ corresponding to the mechanical pain threshold during the first test stimulus and the mechanical pain threshold during the second test stimulus. Negative values indicated an elevation of the threshold, i.e., an inhibitory response due to application of the conditioned test stimulus or more efficient CPM.

### 2.7 Adverse Effects

Adverse effects were evaluated using structured forms that included questions about symptoms related to autonomic stimulation. The questions were categorized into six blocks of possible adverse effect types: (a) gastrointestinal, (b) cardiac, (c) pain, (d) neurological, (e) alterations in ear/nose/throat, and (f) other symptoms. Individuals were asked about these after each intervention session (Redgrave et al. 2018).

### 2.8 Statistical Analyzes

The data were organized in Excel and analyzed using the IBM SPSS Ver. 25 and JASP Ver. 0.18. Descriptive statistics were used to summarize the individual characteristics of BMI, blood pressure, physical activity level, CPM, HRV, and adverse effects. The Shapiro–Wilk test was performed to test the normality of the data. Generalized estimating equations (GEEs) were used to evaluate the impact of interventions (tCMS, iTBS, taVNS, and Sham‐taVNS) on HRV variability, testing the main effects of Time, Group, and the interaction of Time × Group.

Bonferroni correction for multiple comparisons was performed in GEE within each individual outcome. All statistical analyses were performed at a significance level of 5%.

## 3 Results

### 3.1 Individual Characteristics

The study included 22 healthy individuals, half of whom were females, with a mean age of 29.23 ± 12.58 years. Most participants demonstrated high physical activity; the average BMI was 24.83 ± 3.7. The baseline characteristics of the individuals are summarized in Table 1.

**TABLE 1:** Baseline characteristics of the enrolled individuals.

Feature	Statistic
Gender, female, *n* (%)	12 (54.4)
Age, mean years (SD)	29.23 (12.6)
Height, mean cm (SD)	166.55 (11.7)
Weight, mean kg (SD)	69.54 (16.6)
BMI, mean (SD)	24.83 (3.7)
Physical activity level, *n* (%)	
Low	6 (27.3)
Moderated	3 (13.6)
High	13 (59.1)
Relative CPM‐0min, mean (SD)	−6.19 (20.1)
Relative CPM‐5min, mean (SD)	3.47 (18.21)
Mean heart rate, mean (SD)	65.14 (9.4)
Blood pressure, mean (SD)	
Systolic	113.6 (8.1)
Diastolic	72.5 (6.1)
Breath rate, mean (SD)	13.5 (4.4)
HRV, median (IQR)	
rMSSD	40.76 (37.4–75.7)
SDNN	47.64 (33.09–65.18)
Total power HRV	2115.16 (967.7–3792.5)
LF normalized power	40.97 (24.9–73.5)
HF normalized power	59 (26.4–75)
VLF relative power	4.55 (3–6.4)
LF/HF ratio	0.70 (0.3–2.8)

### 3.2 HRV

**FIGURE 2:**
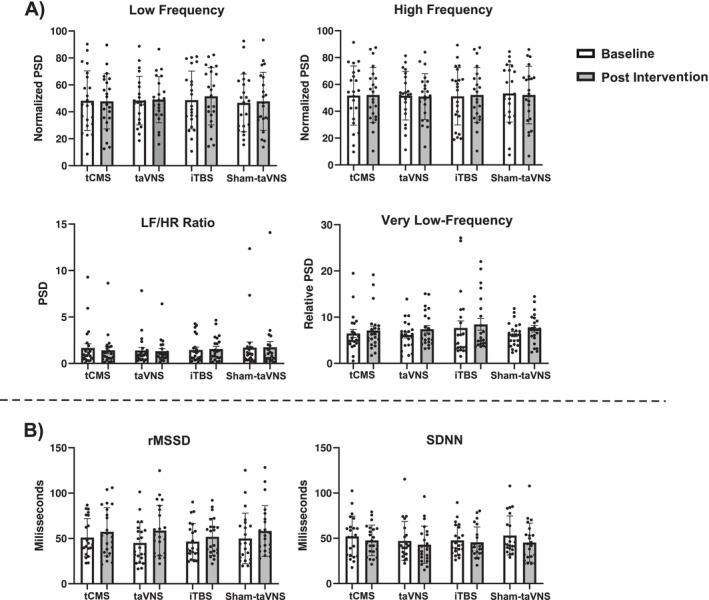
Effect of different intervention techniques on HRV frequency and time domain parameters. Legend: normalized PSD (top row), nominal PSD (middle row), and (B) rMSSD and SDNN (bottom row). Dark and light columns represent baseline and post‐intervention conditions. Error bars represent the standard error of the mean (SEM).

The analysis of HRV in the frequency domain revealed a significant main effect of Time (Wald [1] = 12.95, *p* = 0.000) on Total Power, indicating that the mean Total Power was higher after stimulation compared to baseline. However, no difference was observed in the main effect group (Wald [3] = 2.80, *p* = 0.423) and Time × Group interaction (Wald [3] = 7.01, *p* = 0.072). The same result was observed in the individual frequency band of VLF power spectral in the main effect of Time (Wald [1] = 13.56, *p* = 0.000), Group (Wald [3] = 0.26, *p* = 0.969), and Time × Group interaction (Wald [3] = 3.61, *p* = 0.307). Furthermore, normalized LF, HF, and LF/HF ratio spectral power also presented no statistical difference compared with the control group. Similarly, no significant effects were observed in the time domain measures of rMSSD and SDNN (Figure 2). To confirm these results, the autonomic response to the neuromodulatory effect was tested and also revealed no significant between‐group differences in Total Power (H(3) = 5.18, *p* = 0.159); normalized LF (H(3) = 0.51, *p* = 0.918); and normalized HF (H(3) = 1.45, *p* = 0.693), suggesting that in our study the neuromodulatory intervention was not efficient in inducing autonomic modulation. The effect size (dppc2) (Morris 2008) of neuromodulatory interventions relative to Sham‐taVNS in each HRV measure was calculated and presented in Data S1.

**FIGURE 3:**
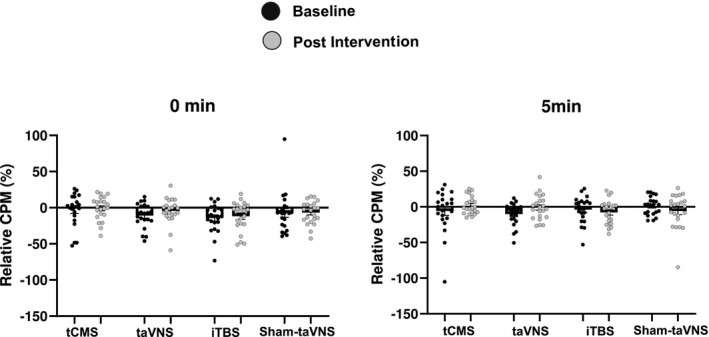
Effect of different neuromodulation interventions on conditioned pain modulation (CPM) responses at two time points: immediately after (0 min, left) and 5 min after (right) the test stimulus. Legend—Relative CPM (%) values are presented for baseline (dark spots) and post‐intervention (light spots) across four intervention groups: transcutaneous cervical magnetic stimulation (tCMS), transcutaneous auricular vagus nerve stimulation (taVNS), intermittent theta burst stimulation (iTBS), and Sham‐taVNS. Negative values indicate pain inhibition. Error bars represent the standard error of the mean (SEM).

The analysis of relative CPM‐0min revealed no significant effects. There was no significant main effect of Time (Wald [1] = 2.856, *p* = 0.091), Group (Wald [3] = 4.305, *p* = 0.230), or Time × Group interaction (Wald [3] = 0.727, *p* = 0.867). Similarly, there were no significant effects in relative CPM‐5min in Time (Wald [1] = 0.057, *p* = 0.504), Group (Wald [3] = 2.798, *p* = 0.424), and Time × Group interaction (Wald [3] = 1.116, *p* = 0.773). These results showed that a single session of autonomic neuromodulatory stimulation could not modulate the descending opioidergic pathway in healthy individuals (Figure 3).

### 3.3 Safety and Adverse Effects of Autonomic Neuromodulation Techniques

Adverse effects following a single session of autonomic neuromodulatory stimulation across the four intervention types (tCMS, iTBS, taVNS, and Sham‐taVNS) are summarized in Table 2. The safety assessment of a single tCMS session revealed fewer adverse effects compared with other autonomic neuromodulatory techniques in all adverse effects categories. **(A) Gastrointestinal:** tCMS presented no adverse effects, whereas isolated cases of vomiting (4.5%) and diarrhea (4.5%) were reported in taVNS and taVNS‐Sham, respectively. **(B) Ear/nose/throat:** tCMS showed only mild tingling (4.55%) and no cases of redness, which were more frequent in taVNS (59.1%) and dlPFC iTBS (45.45%). **(C) Cardiological:** tCMS had one isolated report of hypotension (4.55%), while palpitations were observed in dlPFC iTBS (9.09%), taVNS (4.5%), and taVNS‐Sham (9.1%). **(D) Pain:** Neck pain and stiffness were rare in tCMS (4.55%) compared to higher frequencies in taVNS (9.1%) and dlPFC iTBS (31.82% for pain at the stimulation site). **(E) Neurological:** The somnolence was the most common adverse effect in tCMS (18.18%) but occurred at higher rates in taVNS (40.9%) and taVNS‐Sham (40.9%). **(F) Other symptoms:** tCMS exhibited no cases of fatigue, dizziness, or other non‐specific symptoms, which were more prominent in taVNS (18.2% fatigue) and dlPFC iTBS (13.64% seasickness).

**TABLE 2:** Participants presenting adverse effects of a single session of autonomic neuromodulatory stimulation (*N* (%)).

Adverse effect	Sham‐taVNS	taVNS	iTBS	tCMS
Gastrointestinal				
Strange feeling in the stomach	—	3 (13.64)	—	—
The feeling of wanting to go to the bathroom	—	—	2 (9.09)	—
Vomiting	—	1 (4.55)	—	—
Diarrhea	1 (4.55)	—	—	—
Local				
Redness	9 (40.9)	13 (59.1)	10 (45.45)	—
Tingling	10 (45.45)	10 (45.45)	7 (31.82)	1 (4.55)
Itch	4 (18.18)	5 (22.7)	4 (18.18)	—
Ear, nose, and throat				
Buzz	—	1 (4.55)	1 (4.55)	—
Difficulty hearing sounds	—	—	—	1 (4.55)
Vertigo	1 (4.55)	1 (4.55)	—	—
Cardiological				
Palpitation	2 (9.09)	1 (4.55)	2 (9.09)	—
Hypotension	—	—	—	1 (4.55)
Pain				
Pain at the stimulation site	1 (4.55)	7 (31.82)	7 (31.82)	—
Neck pain	1 (4.55)	2 (9.09)	—	1 (4.55)
Pain teeth	1 (4.55)	1 (4.55)	2 (9.09)	—
Chest pain	—	—	2 (9.09)	—
Pain shoulder	—	2 (9.09)	—	—
Joint pain	—	2 (9.09)	—	—
Neurological				
Somnolence	9 (40.9)	9 (40.9)	6 (27.27)	4 (18.18)
Involuntary contraction in the mouth	—	—	11 (50.00)	1 (4.55)
Involuntary eye twitching	—	—	8 (36.36)	—
Headache	—	2 (9.09)	4 (18.18)	1 (4.55)
Microshocks	—	1 (4.55)	—	—
Metallic taste	1 (4.55)	—	—	—
Other symptoms				
Seasickness	1 (4.55)	4 (18.18)	3 (13.64)	—
Fatigue	—	4 (18.18)	—	—
Neck spasm	—	1 (4.55)	2 (9.09)	1 (4.55)
Stiff neck	—	2 (9.09)	2 (9.09)	—
Dizziness	1 (4.55)	3 (13.64)	—	—
Shortness of breath	1 (4.55)	—	—	—
Urinary frequency	1 (4.55)	—	—	—

## 4 Discussion

This study assessed the effectiveness and safety of a single tCMS session for modulating parasympathetic activity and opioid endogenous modulation in healthy individuals in comparison with iTBS and taVNS. To the best of our knowledge, this is the first study to directly compare these techniques in a healthy population. Our findings demonstrate that neither of the techniques was superior to Sham stimulation in modulating HRV or CPM. Nevertheless, tCMS was found to be a well‐tolerated, safe intervention, associated with fewer adverse effects compared to both taVNS and iTBS.

### 4.1 Effectiveness of Neuromodulation in HRV and CPM

The results suggest that the neuromodulatory effect of the tCMS on the autonomic neuromodulatory effect is similar to that of the other techniques. No difference was found among the studied techniques and Sham‐taVNS in HRV and relative CPM. The absence of significant changes in HRV across groups suggests that a single session may not be sufficient to induce measurable autonomic modulation, which aligns with prior data indicating that repeated stimulation is often required to achieve consistent effects (Le Roy et al. 2023). However, a study that treated depression using 10 consecutive sessions of iTBS had neither negative nor positive effects on HRV. The lack of impact on relative CPM contrasts with previous studies demonstrating that a single session of vagal stimulation can increase experimental pain perception thresholds (Busch et al. 2013; Pacheco‐Barrios et al. 2024). A possible explanation for this discrepancy is a shorter stimulation duration (30 min) in our study. In comparison, prior research conducted in both clinical and experimental settings employed 60 min of stimulation (Aoyagi et al. 2025; Busch et al. 2013; Pacheco‐Barrios et al. 2024). This difference in stimulation parameters could account for the reduced responsiveness of endogenous pain modulation pathways to acute neuromodulatory interventions in healthy individuals.

### 4.2 Safety Profile of tCMS

The safety profile of tCMS observed in this study aligns with emerging evidence from prior research. For instance, a recent study investigating tCMS targeting the cervical region reported that five out of 10 participants experienced neck stiffness, which was alleviated by movement (H. Zhang, Zhao, et al. 2024). Other clinical studies, including a systematic review, have shown that in patients with migraine, adverse effects included discomfort and nasopharyngitis, along with rarer occurrences of influenza, oropharyngeal pain, dizziness, application site reactions (rash and erythema), and ear discomfort, all affecting fewer than 9.5% of participants (Diener et al. 2019; Lai et al. 2020; Tassorelli et al. 2018). The auricular branch of the vagus nerve provides another pathway for stimulation. A systematic review of 177 studies on the safety of taVNS revealed that more than half did not report any adverse events. Among the 35 studies that did, a meta‐analysis showed no significant difference in the risk of adverse effects between taVNS and control groups. The most commonly reported adverse effects included ear pain, headache, and tingling, with no causal relationship established between taVNS and severe adverse events (Kim et al. 2022). Similarly, no severe adverse effects were observed in our study. However, participants did report isolated cases of vomiting, palpitations, and vertigo, and some cases of pain, neck stiffness, seasickness, itch, fatigue, and somnolence, among others. Tingling and redness were the most common adverse effects. Left DLPFC iTBS is being studied mostly in psychiatric diseases, and its adverse effects are still scarce in the literature. A systematic review of this technique applied in patients with schizophrenia reported mostly mild effects unrelated to schizophrenia, such as fatigue, headache, neck pain, and hypertension. However, there was one report of a severe adverse effect, a seizure episode during the iTBS application (Poorganji et al. 2023). Other studies reported headache as the most common adverse effect (Blumberger et al. 2018; Chen et al. 2020; M. Zhang, Li, et al. 2024). Accordingly, no severe adverse effect was found in our study, and volunteers mostly reported involuntary mouth contraction, eye contraction, and redness. Among the studied techniques, tCMS was the one with the fewest adverse events reported. These findings show the favorable safety profile of tCMS while emphasizing the importance of systematically evaluating adverse effects across different neuromodulatory techniques to ensure safe and effective applications.

### 4.3 Limitations

Some limitations of this study should be acknowledged. First, the small sample size may limit the generalizability of the findings, particularly given the variability in individual responses to neuromodulation. Second, the Sham control was limited to taVNS, which may introduce bias, as the Sham condition did not mimic all interventions. Future studies should employ more robust Sham controls that account for the sensory and physiological effects of each technique. Third, HRV measurements were conducted with participants lying down and eyes closed, which may have increased parasympathetic activity due to relaxation or somnolence (Tobaldini et al. 2017; Trinder et al. 2001). In the current study, the prevalence of somnolence was relatively high across all intervention groups, which could mask real differences between groups and serve as a potential source of bias. Alternative measurement conditions (e.g., seated, eyes open) should be considered in future studies to minimize this potential confounder. Finally, although our findings do not support differential efficacy among the tested techniques, tCMS was well tolerated and associated with fewer adverse effects in healthy participants exposed to a single stimulation session, supporting its safety profile for further research. Therefore, further research is needed to determine whether similar adverse effects can be observed in populations with different clinical conditions and with a larger number of sessions. Such studies may increase our understanding of the role of tCMS in HRV and relative CPM.

## 5 Conclusion

In conclusion, the findings demonstrate that a single session of tCMS is a safe and feasible intervention, with fewer adverse effects compared to taVNS and iTBS. However, the lack of significant changes in HRV and CPM suggests that acute neuromodulation may not be sufficient to induce measurable autonomic or pain modulation in healthy individuals. Future studies should investigate the effects of repeated tCMS sessions, longer stimulation durations, and alternative measurement conditions to better understand its therapeutic potential. Additionally, research in clinical populations (e.g., patients with chronic pain and autonomic dysfunction) is needed to evaluate the efficacy of tCMS in modulating autonomic activity and pain pathways. By addressing these gaps, future research can further elucidate the role of tCMS in non‐invasive neuromodulation and its potential applications in clinical practice.

## Author Contributions

**Tiago da Silva Lopes:** conceptualization, data curation, formal analysis, investigation, visualization, writing – original draft, writing – review and editing. **Larissa Conceição Dias Lopes:** conceptualization, data curation, formal analysis, visualization, writing – original draft, writing – review and editing. **Thaiany Pedrozo Campos Antunes:** conceptualization, writing – original draft, writing – review and editing. **Isabela Rocha Fernandes:** conceptualization, investigation, writing – original draft, writing – review and editing. **Márcia Midori Morimoto:** conceptualization, investigation, writing – original draft, writing – review and editing. **Gustavo Pinheiro:** conceptualization, investigation, writing – original draft, writing – review and editing. **Erick Dario León Bueno de Camargo:** conceptualization, writing – original draft, writing – review and editing. **Bruna Ferreira Nonato:** conceptualization, investigation, writing – original draft, writing – review and editing. **Maria Sophia Cantisani:** conceptualization, investigation, writing – original draft, writing – review and editing. **Karina Rabello Casali:** conceptualization, data curation, formal analysis, writing – original draft, writing – review and editing. **Pedro Montoya:** conceptualization, writing – original draft, writing – review and editing. **Yossi Zana:** conceptualization, data curation, formal analysis, methodology, supervision, writing – original draft, writing – review and editing. **Abrahão Fontes Baptista:** conceptualization, methodology, supervision, writing – original draft, writing – review and editing.

## Funding

T.S.L. acknowledges receiving a postdoctoral grant from CAPES (Process Number: 88887.801519/2023‐00). L.C.D.L. received a PhD grant from CAPES during the study development (Process Number: 88887.939716/2024‐00). T.P.C.A. received a postdoctoral grant from CAPES (Process Number: 88887.976525/2024‐00). A.F.B. acknowledges receiving a grant for scientific productivity from CNPq (Brazil) (Process Number: 306959/2025‐9). P.M. received grants PID2022‐140561NB‐I00 funded by MICIU/AEI/10.13039/501100011033 and by “ERDF A way of making Europe” by “ERDF/EU”, and #2022/12916‐9 funded by São Paulo Research Foundation (FAPESP). G.P. received a master's degree grant from CAPES—Finance Code 001.

## Conflicts of Interest

The authors declare no conflicts of interest.

## Supporting information


**Data S1:** Supporting information.

## Data Availability

The database and codes for this study can be obtained upon request from the corresponding author.
