# Supplementary material for: Feasibility and Safety of a Single‐Session of Transcutaneous Cervical Magnetic Stimulation, taVNS, and iTBS on Heart Rate Variability, Safety, and Pain Modulation
Source: Eur J Neurosci. 2025 Dec 27;62(12):e70370. doi: 10.1111/ejn.70370 (PMC12743293; doi:10.1111/ejn.70370)
Supplement: Supplementary file 1 — Data S1: Supporting information. [file EJN-62-0-s001.docx]

| **Effect-size of neuromodulatory interventions relative to taVNS Sham condition in each HRV measures** | | | | | |
| --- | --- | --- | --- | --- | --- |
|  |  |  | **Sham-taVNS** | |  |
|  | **Baseline Mean (SD)** | **Post Intervention Mean (SD)** | **Baseline Mean (SD)** | **Post Intervention Mean (SD)** | **Effect Size_dppc2_** |
| **Low Frequency HRV** | | | | | |
| **tCMS** | 48.23 (22.14) | 47.78 (20.38) | 46.41 (21.39) | 47.83 (21.51) | - 0.08 |
| **taVNS** | 48.34 (18.03) | 49.01 (17.15) |  |  | - 0.04 |
| **iTBS** | 48.73 (21.43) | 51.58 (21.25) |  |  | 0.07 |
| **High Frequency HRV** | | | | | |
| **tCMS** | 51.68 (22.10) | 52.16 (20.38) | 53.31 (21.38) | 52.11 (21.48) | 0.08 |
| **taVNS** | 51.58 (18.01) | 50.93 (17.14) |  |  | 0.03 |
| **iTBS** | 51.16 (21.37) | 48.36 (21.24) |  |  | - 0.07 |
| **LF/HR Ratio** | | | | | |
| **tCMS** | 1.67 (2.18) | 1.42 (1.77) | 1.71 (2.80) | 1.75 (2.92) | - 0.11 |
| **taVNS** | 1.4 (1.66) | 1.31 (1.31) |  |  | - 0.05 |
| **iTBS** | 1.46 (1.37) | 1.56 (1.30) |  |  | 0.03 |
| **Very Low Frequency** | | | | | |
| **tCMS** | 6.46 (4.16) | 7.11 (4.41) | 6.33 (2.64) | 7.49 (3.33) | - 0.14 |
| **taVNS** | 6.14 (3.05) | 7.39 (3.76) |  |  | 0.03 |
| **iTBS** | 7.66 (7.21) | 8.45 (5.89) |  |  | - 0.07 |
| **rMSSD** | | | | | |
| **tCMS** | 50.93 (21.06) | 47.66 (16.87) | 49.97 (27.91) | 58.23 (27.97) | - 0.46 |
| **taVNS** | 45.04 (22.47) | 58.65 (27.98) |  |  | 0.21 |
| **iTBS** | 46.62 (19.67) | 51.81 (20.44) |  |  | - 0.12 |
| **SDNN** | | | | | |
| **tCMS** | 52.06 (22,32) | 47.66 (16.87) | 53.03 (21.60) | 45.25 (21.47) | 0.15 |
| **taVNS** | 47.11 (21.44) | 42.91 (20.51) |  |  | 0.16 |
| **iTBS** | 47.72 (16.70) | 45.51 (17.01) |  |  | 0.29 |
